# Comparing the association between out‐of‐pocket cost burden and cost‐related care avoidance among individuals with and without a history of cancer

**DOI:** 10.1002/cncr.70495

**Published:** 2026-06-15

**Authors:** Xi Liang, Djin Tay, Yu Ke, Ding Quan Ng, Chanthawat Patikorn, Carl Asche, Nathorn Chaiyakunapruk, Anne C. Kirchhoff, Raymond Javan Chan, Alexandre Chan, Chia Jie Tan

**Affiliations:** ^1^ Department of Pharmacotherapy College of Pharmacy University of Utah Salt Lake City Utah USA; ^2^ College of Nursing University of Utah Salt Lake City Utah USA; ^3^ Cancer Control and Population Sciences Program Huntsman Cancer Institute Salt Lake City Utah USA; ^4^ Division of Supportive and Palliative Care National Cancer Centre Singapore Singapore; ^5^ School of Pharmacy & Pharmaceutical Sciences University of California Irvine Irvine California USA; ^6^ Department of Social and Administrative Pharmacy Faculty of Pharmaceutical Sciences Chulalongkorn University Bangkok Thailand; ^7^ IDEAS Center, Veterans Affairs Salt Lake City Healthcare System Salt Lake City Utah USA; ^8^ Division of Pediatric Hematology and Oncology Department of Pediatrics School of Medicine University of Utah Salt Lake City Utah USA; ^9^ Caring Futures Institute College of Nursing and Health Sciences Flinders University Adelaide South Australia Australia

**Keywords:** out‐of‐pocket costs, household income, financial toxicity, care avoidance, predictive model

## Abstract

**Introduction:**

Out‐of‐pocket (OOP) cost is routinely captured by payers and health systems and may help identify individuals at risk of cost‐related care avoidance. This study aimed to evaluate the relationship between OOP cost burden relative to household income (OOP:HHI) and cost‐related care avoidance and to assess whether this relationship differs by cancer history.

**Methods:**

A retrospective cross‐sectional study was conducted leveraging data from multiple survey waves of the Understanding America Study (2015–2024). Adults aged ≥18 years with data on OOP health care expenditure, household income, and cost‐related care avoidance were included. Mixed‐effects logistic models with random intercepts were used to assess associations between OOP:HHI and care avoidance, adjusting for respondent characteristics. Effect modification by cancer history was examined. Classification performance of OOP:HHI alone versus multivariable models was evaluated using receiver operating characteristic curves.

**Results:**

The analysis included 21,299 responses from 10,811 respondents, of which 2180 responses were from 1052 respondents with cancer history. Higher OOP:HHI was independently associated with increased odds of cost‐related care avoidance (adjusted odds ratio per 1% increase = 1.03; 95% CI, 1.02–1.04) with similar trends in both cancer and noncancer subgroups. OOP:HHI alone showed modest discrimination, whereas multivariable models demonstrated excellent performance in identifying respondents who indicated care avoidance.

**Conclusion:**

Higher OOP cost burden was associated with cost‐related care avoidance regardless of cancer history, but cost burden alone was insufficient for risk identification. Integrating OOP costs with routinely available patient characteristics may better identify individuals at risk of care avoidance for targeted interventions to mitigate financial toxicity.

## Abstract 2

Accounting for household income, higher out‐of‐pocket cost burden was associated with greater odds of cost‐related care avoidance with similar trends among individuals with and without cancer history. Out‐of‐pocket cost burden alone showed limited ability to identify individuals at risk of cost‐related care avoidance but incorporating other patient characteristics routinely collected by health systems demonstrated substantially higher classification performance.

## INTRODUCTION

Financial toxicity refers to the adverse economic consequences of medical care for patients and their families.^1^ Because of the high cost, extended duration, and reduced productivity associated with cancer treatment, financial toxicity is well‐documented among individuals with cancer history, including those who have completed primary tumor‐directed therapy.^2^, ^3^, ^4^, ^5^ Among the most severe consequences of financial toxicity is cost‐related care avoidance, in which patients forego necessary medical care because they are unable to afford it. This results in suboptimal antineoplastic treatment, inadequate monitoring or surveillance of cancer, and poor control of other comorbidities. Identifying individuals at risk of cost‐related care avoidance is critical to allow early implementation of interventions to mitigate financial toxicity, such as insurance navigation, financial counseling, and copay assistance programs.^6^, ^7^, ^8^, ^9^ Identification of at‐risk patients is particularly important in health systems with substantial cost‐sharing, such as the United States, where high deductibles, coinsurance, and copayments can impose significant financial strain, even among insured patients.^2^

Practical tools in the clinical setting to identify individuals at risk of cost‐related cost avoidance remain limited.^6^ Given that out‐of‐pocket (OOP) health care expenditure is routinely collected by payers and health systems as part of billing records, increasing OOP cost burden may readily serve as an early indicator of patients at risk of cost‐related care avoidance. Although thresholds of 10%, 25%, and 40% of household income (HHI) for OOP health care expenditure have been used by the World Bank and the World Health Organization to indicate catastrophic health expenditure, these thresholds are primarily policy evaluation tools based on economic theory and have not been empirically validated to predict behavioral outcomes such as care avoidance.^10^, ^11^ Additionally, the same thresholds are applied across different disease areas and patient populations without considering that the price elasticity of health care demand may vary by health conditions.^10^, ^11^ Individuals with cancer history, for example, may be willing to tolerate a higher OOP cost burden because of their prior experience with a potentially life‐threatening condition and their perceived importance of ongoing care. In contrast, it may also be possible that persistent long‐term financial strain after cancer treatment reduces the ability of cancer survivors to absorb additional health care expenses, increasing the likelihood to forgo medical care, even at relatively modest levels of cost burden.

Although many studies have assessed OOP cost burden in the oncology population, most do not account for the capacity to pay, such as HHI, and very few have assessed the relationship between OOP cost and health‐seeking behavior.^12^ As a result, little is known about how specific levels of OOP cost burden translate into behavioral responses such as care avoidance. Additionally, whether individuals with and without cancer history respond similarly to escalating OOP costs remains unclear. Understanding these differences is important given the rapid growth in the cancer survivor population and the rising cost of cancer care.

The aims of this study were therefore (1) to quantify the relationship between OOP cost (accounting for the capacity to pay) and the likelihood of cost‐related care avoidance and (2) to assess if similar trends of association between OOP cost burden and care avoidance are observed among individuals with and without cancer history. We also investigated whether OOP cost burden, measured relative to capacity to pay, can serve as an effective predictor for patients at high risk for cost‐related care avoidance. Our findings will inform the potential role of OOP cost burden as an accessible, system‐level tool to identify individuals at an elevated risk of forgoing necessary medical care. Such insights may support more targeted allocation of financial navigation resources and contribute to proactive strategies to mitigate long‐term financial toxicity among cancer survivors.

## MATERIALS AND METHODS

### Study design, data source, and study population

We conducted a retrospective cross‐sectional study leveraging publicly available data from the Understanding America Study (UAS). UAS is a probability‐based internet panel based at the University of Southern California and includes approximately 15,000 U.S. residents. Longitudinal surveys fielded by the UAS cover a wide range of topics involving health, income, wealth, and financial literacy, with both English and Spanish versions. UAS participants regularly complete surveys online, and the study provides internet access and devices to those without them to ensure inclusive representation.^13^ The survey dataset is deidentified and includes rich data that are widely used in social science and policy research to analyze trends, behavioral patterns, and population‐level responses over time. Respondents who were aged 18 years or older, with available responses on OOP health care expenditure, HHI, and the inability to afford healthcare services when required were eligible for our study. All responses by eligible respondents across all survey waves were included regardless of cancer history to assess the impact of cancer history on the relationship between OOP cost burden and cost‐related care avoidance. Respondents were considered to have cancer history if they have ever been told by a doctor that they have been diagnosed with cancer (excluding nonmelanoma skin cancer). Respondents included in the cancer subgroup may have been living with cancer or were in cancer remission, aligning with the definition of cancer survivors by the National Cancer Institute, which is considered to start from the time of cancer diagnosis through the balance of life.^14^

### Study variables

The exposure variable of interest was the percentage of HHI used for OOP health care expenditure (OOP:HHI) to account for the capacity to pay. OOP health care expenditure was obtained from self‐reported out‐of‐pocket payments in a range of categories, such as doctor visits, hospital care, outpatient surgery, prescription medications, dental care, nursing home care, in‐home health care, and other medical services that were not covered by insurance. This would include copayments, coinsurance, deductibles, and payments for noncovered services. In the UAS, respondents reported HHI as categories. In our analysis, we used the midpoint of each income range to approximate HHI for each respondent. The recall period for both OOP and HHI was 2 years.

The outcome variable of interest was cost‐related care avoidance, which is defined as the inability to afford healthcare services when required. Respondents were specifically probed if there was any time during the past 2 years before the survey when they needed medical care but did not get it because they could not afford it. Respondents who provided a positive response to this question were considered to have exhibited cost‐related care avoidance.

Other variables, including demographics (age, gender, race, ethnicity, marital status, family size, and health insurance), socioeconomic status (education, employment status), and comorbidity history (hypertension, diabetes, chronic lung disease, heart conditions, arthritis, and mental health conditions), were also identified from survey responses. For comorbidity history, respondents were considered to have a history of a specific comorbidity if they indicated that they have ever been told by a doctor that they have the health condition, whether in the current or a previous survey.

### Statistical analysis

The baseline characteristics of the respondents were reported descriptively based on the most recent survey of each respondent, stratified by cancer history. Categorical data were presented as counts and percentages, and continuous data were presented as mean (standard deviation) or median (interquartile range). The proportions of respondents who reported cost‐related care avoidance were stratified by OOP:HHI thresholds of 10%, 25%, and 40% (conventional thresholds to indicate catastrophic health care expenditure) and cancer history. The distribution of OOP health care expenditure by cost component was also described qualitatively for both individuals with and without cancer history.

To assess the association between OOP:HHI and cost‐related care avoidance, a mixed‐effects logistic regression model with random intercepts was used to account for within‐subject correlation as the same respondents could be represented more than once in multiple survey waves. OOP:HHI was modeled as a continuous variable (in %) and as a sensitivity analysis to assess the robustness of the association, OOP:HHI was also modeled as a categorical variable using cutoffs of 10%, 25%, and 40%. Univariable analysis was performed first, followed by multivariable analysis adjusting for covariates, including demographics, socioeconomic status, and other comorbidities, which were statistically significant in univariable analyses. An interaction term between cancer history and OOP:HHI was also incorporated to examine potential effect modification by cancer history on the relationship between OOP:HHI and care‐related care avoidance. The marginal effect of cancer history on the likelihood of cost‐related care avoidance across a range of OOP:HHI values was also visualized.

The performance of two different methods in identifying respondents with cost‐related care avoidance was compared: (1) OOP:HHI alone and (2) predicted probabilities from the multivariable logistic regression model generated previously, which incorporated OOP:HHI and other respondent characteristics. The optimal thresholds for both methods were identified using an approach proposed by Liu et al.,^15^ which aimed to maximize the product of sensitivity and specificity for each method. Classification performance was then evaluated by plotting receiver operating characteristic (ROC) curves for both the cancer and noncancer subgroups. A higher area under the curve (AUC) indicated better performance. All statistical tests were two‐sided, with significance defined as a *p* value < .05. All data analyses were conducted using STATA version 19.0 (StataCorp).

## RESULTS

### Baseline characteristics

Our analysis included 21,299 responses from a total of 10,811 respondents between 2015 and 2024 across five survey waves, with 2180 responses from 1052 respondents after reporting cancer history and 19,119 responses from 9759 respondents without cancer history. The cancer subgroup was older than the noncancer subgroup (median age 67 vs. 49 years), whereas both cancer and noncancer subgroups were predominantly female (59.2% and 60.8%), White (83.3% and 76.9%), non‐Hispanic/Latino (93.2% and 84.6%), and received at least higher education (59.3% and 59.4%). Compared to the noncancer subgroup, the cancer subgroup comprised a higher proportion of individuals who were not working (64.7% vs. 35.9%) and a lower proportion of respondents with private employment (44.9% vs. 21.9%). Commercial insurance coverage was common in both groups (58.5% and 67.4%), but public insurance coverage (especially Medicare) was substantially higher among respondents with cancer history (67.2% vs. 33.2%). The cancer subgroup had a lower proportion of respondents without any insurance coverage (either public or commercial) compared with the noncancer subgroup (3.8% vs. 10.6%). Respondents in the cancer subgroup also reported a higher prevalence of comorbidities compared to the noncancer subgroup, including hypertension (58.7% vs. 38.1%), diabetes (27.2% vs. 15.8%), chronic lung disease (13.3% vs. 4.9%), and mental health conditions (33.8% vs. 32.4%) (Table 1).

**TABLE 1:** Characteristics of eligible participants stratified by cancer history.

Patient characteristics^a^		Cancer subgroup^b^ (*N* = 1052)	Noncancer subgroup (*N* = 9759)
Age at survey, median (Q1, Q3)		67 (56, 74)	49 (36, 62)
Gender	Female	623 (59.2%)	5,929 (60.8%)
	Male	429 (40.8%)	3,829 (39.2%)
	Unreported	0 (0%)	1 (<1%)
Race	Asian and Pacific Islander only	33 (3.1%)	623 (6.4%)
	American Indian or Alaska Native only	15 (1.4%)	160 (1.6%)
	Black or African American only	74 (7.0%)	852 (8.7%)
	White only	876 (83.3%)	7,508 (76.9%)
	Mixed	52 (4.9%)	552 (5.7%)
	Unreported	2 (<1%)	64 (<1%)
Ethnicity (Spanish/Hispanic/Latino)	Yes	71 (6.7%)	1,500 (15.4%)
	No	980 (93.2%)	8,259 (84.6%)
	Unreported	1 (<1%)	0 (0%)
Highest education attainment	High school or lower	428 (40.7%)	3,967 (40.6%)
	Attended college or higher	624 (59.3%)	5,792 (59.4%)
	Unreported	0 (0%)	0 (0%)
Annual household income	<$20,000	135 (12.8%)	1165 (11.9%)
	$20,000 to <$40,000	204 (19.4%)	1647 (16.9%)
	$40,000 to <$60,000	180 (17.1%)	1464 (15.0%)
	$60,000 to <$100,000	248 (23.6%)	2426 (24.9%)
	$100,000 to <$150,000	151 (14.4%)	1590 (16.3%)
	$150,000 and higher	134 (12.7%)	1467 (15.0%)
Marital status	With partners(s)	615 (58.5%)	5419 (55.5%)
	Without partners(s)	437 (41.5%)	4338 (44.5%)
	Unreported	0 (0%)	2 (<1%)
Employment status^c^	Government	70 (6.7%)	1152 (11.8%)
	Private	230 (21.9%)	4386 (44.9%)
	Self‐employed	71 (6.7%)	711 (7.3%)
	Not working	681 (64.7%)	3503 (35.9%)
	Unreported	0 (0%)	7 (<1%)
Family size, median (Q1, Q3)		2 (2, 2)	2 (2, 4)
Public health insurance^d^ ^,^ ^e^	Covered	707 (67.2%)	3243 (33.2%)
	Medicaid alone^f^	46 (6.5%)	747 (23.0%)
	Medicare alone^f^	540 (76.4%)	1852 (57.1%)
	Medicaid and Medicare^f^	62 (8.8%)	353 (10.9%)
	Other public plan^f^	59 (8.3%)	291 (9.0%)
	Not covered	345 (32.8%)	6516 (66.8%)
	Unreported	0 (0%)	0 (0%)
Commercial health insurance^e^	Covered	615 (58.5%)	6576 (67.4%)
	Not covered	436 (41.4%)	3147 (32.2%)
	Unreported	1 (<1%)	36 (<1%)
Hypertension	Yes	618 (58.7%)	3717 (38.1%)
	No	434 (41.3%)	6042 (61.9%)
Diabetes	Yes	286 (27.2%)	1539 (15.8%)
	No	766 (72.8%)	8220 (84.2%)
Chronic lung disease	Yes	140 (13.3%)	474 (4.9%)
	No	912 (86.7%)	9285 (95.1%)
Heart conditions	Yes	257 (24.4%)	947 (9.7%)
	No	795 (75.6%)	8812 (90.3%)
Arthritis	Yes	540 (51.3%)	2544 (26.1%)
	No	512 (48.7%)	7215 (73.9%)
Mental health conditions	Yes	356 (33.8%)	3161 (32.4%)
	No	696 (66.2%)	6598 (67.6%)

*Note*: Data presented as *n* (%), unless otherwise indicated.

^a^ Data based on responses from the most recent survey completed by respondents.

^b^ Cancer subgroup included respondents who were previously told by a doctor that they had cancer, except nonmelanoma skin cancer.

^c^ Not working included respondents who were unemployed, retired, or on disability benefits.

^d^ Public health insurance included Medicaid, Medicare and military plans (e.g., TRI‐CARE, CHAMPUS, CHAMP‐VA).

^e^ Respondents may have both public and private health plans.

^f^ Percentages for subcategories of public health insurance were calculated among respondents covered by public health insurance.

### Patterns of OOP spending and cost‐related care avoidance

**FIGURE 1:**
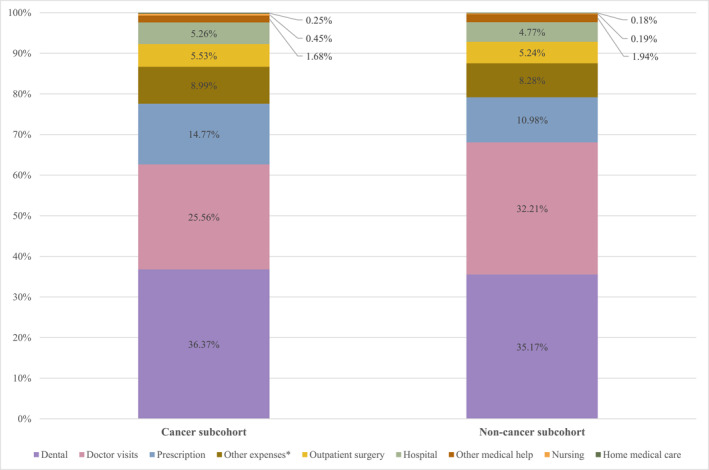
Distribution of OOP by expenditure category stratified by cancer history. Other expenses include expenses not covered under any of the listed categories. OOP indicates out of pocket.

Dental care, doctor visits, and prescription medications were the top contributors of OOP health care expenditure in both the cancer and noncancer subgroups. Responses from individuals with cancer history indicated a higher amount of expenses on prescription medications (14.8% vs. 11.0%) and hospital care (5.3% vs. 4.8%), compared to those from individuals without cancer history (Figure 1). Overall, most responses (*n* = 13,112/21,299; 61.6%) indicated relatively low OOP:HHI (<1%) with only a small proportion (*n* = 927/21,299; 4.4%) with OOP:HHI exceeding the conventional thresholds for catastrophic health care expenditure (≥10%). A slightly higher proportion of responses from individuals with cancer history (*n* = 111/2180; 5.1%) reported OOP:HHI ≥10% compared to responses from those without cancer history (*n* = 819/19,119; 4.3%) (Supplemental Table 1).

**FIGURE 2:**
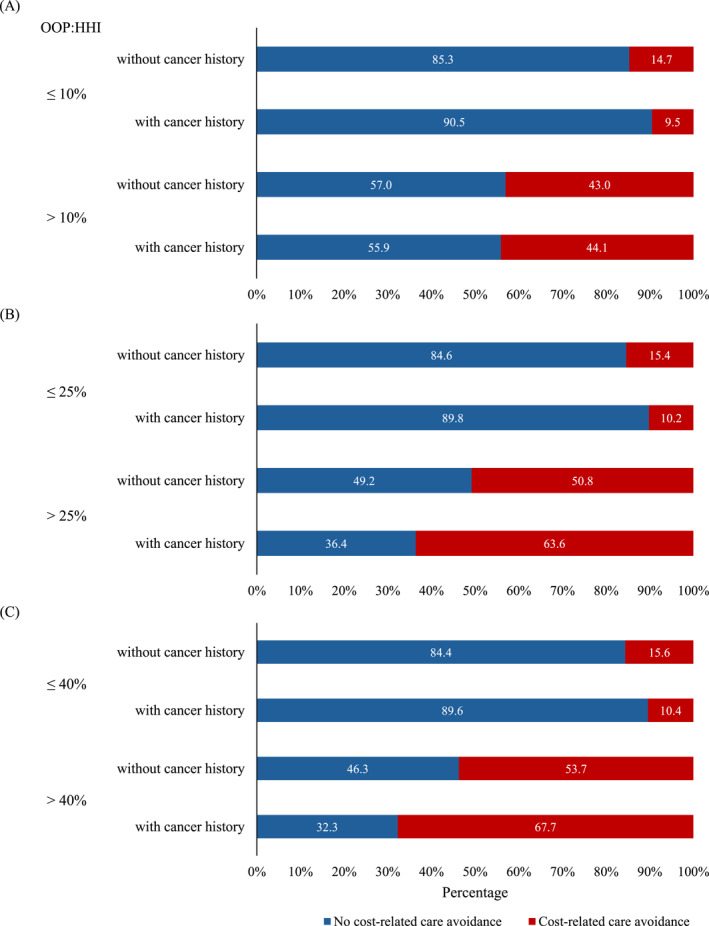
Proportion of patients indicating cost‐related care avoidance stratified by cancer history and OOP:HHI using 10%, 25%, and 40% as thresholds. OOP:HHI indicates out‐of‐pocket cost burden relative to household income.

Among all responses, 3284/21,299 (15.4%) indicated cost‐related care avoidance, whereas 2403/10,811 (22.2%) of all respondents indicated cost‐related care avoidance in at least one survey wave that they participated in. When stratified by cancer history, the proportion of responses indicating cost‐related care avoidance was higher among those without cancer history (*n* = 3309/19,119; 15.9%) than those with cancer history (*n* = 245/2180; 11.2%). Within the cancer subgroup, the proportion of responses reporting cost‐related care avoidance remained low and relatively stable at or below OOP:HHI thresholds 10%, 25%, and 40% but increased sharply once the threshold was exceeded. Specifically, among responses that reported OOP:HHI ≤10%, 9.5% in the cancer subgroup indicated cost‐related care avoidance, whereas among those that reported OOP:HHI >10%, 44.1% indicated cost‐related care avoidance (Figure 2A). As the OOP:HHI thresholds increased, 10.2% and 63.6% of responses below and above 25% indicated cost‐related care avoidance, respectively, whereas 10.4% and 67.7% of responses below and above 40% indicated cost‐related care avoidance, respectively (Figure 2B and Figure 2C). The noncancer subgroup showed similar patterns, although the absolute proportions of responses under the OOP:HHI thresholds indicating cost‐related care avoidance were generally higher than those in the cancer subgroup, whereas those above the thresholds were lower (Figure 2).

### Association between OOP:HHI and cost‐related care avoidance

**FIGURE 3:**
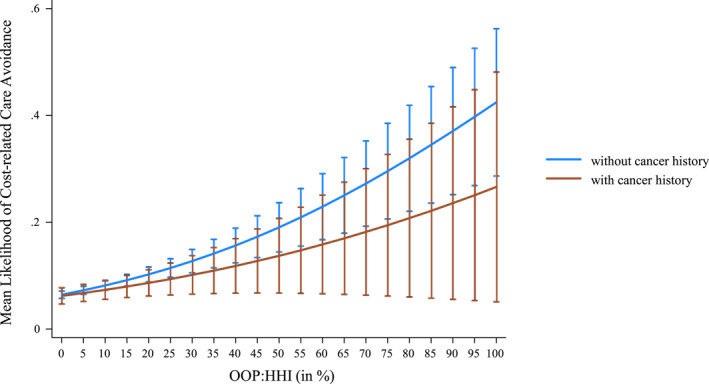
Marginal effect of cancer history on mean likelihood of cost‐related care avoidance across OOP:HHI values. OOP:HHI indicates out‐of‐pocket cost burden relative to household income.

After adjusting for covariates, each 1% increase in OOP:HHI was associated with a 3% higher odds of cost‐related care avoidance (odds ratio = 1.03; 95% CI, 1.02–1.04; *p* < .001) (Table 2). As OOP cost burden increased, the marginal likelihood of cost‐related care avoidance rose for both subgroups, but more sharply for individuals without cancer history (Figure 3). Although cancer history significantly influenced the relationship between OOP:HHI and cost‐related care avoidance in the unadjusted model (*p* = .001), the interaction effect was no longer significant (*p* = .411) in the multivariable model (Table 2). Similar trends were observed in our sensitivity analyses when modeling OOP:HHI as a categorical variable, when including only respondents aged <65 years, or after adjusting for survey year. (Supplemental Table 2‐6).

**TABLE 2:** Association between OOP:HHI (in % as a continuous variable) and cost‐related care avoidance (*N* = 10,811).

Variables	Unadjusted (univariable) analysis^a^				Adjusted (multivariable) analysis^a^ ^,^ ^b^			
					Responses = 20,533			
	Responses	Odds ratio	95% CI	*p* value	Odds ratio		95% CI	*p* value
OOP:HHI (in %)	21,299	1.03	1.03–1.04	<.001	1.03		1.02–1.04	<.001
Cancer (reference ‐ without cancer history)	21,299							
With cancer history		0.49	0.37–0.64	<.001	0.81		0.61–1.08	.152
Cancer interaction (reference ‐ without cancer history)	21,299							
With cancer history		0.98	0.97–0.99	.001	0.99		0.98–1.01	.411
Age at survey	21,278	0.94	0.94–0.95	<.001	0.96		0.95–0.96	<.001
Gender (reference ‐ female)	21,295							
Male		0.33	0.28–0.39	<.001	0.55		0.47–0.64	<.001
Race (reference ‐ White only)	21,198			<.001				<.001
Black only		1.90	1.43–2.51	<.001	1.12		0.86–1.46	.400
American Indian or Alaska Native only		2.59	1.45–4.63	.001	1.25		0.73–2.15	.410
Asian and Pacific Islander only		0.85	0.60–1.22	.383	0.82		0.58–1.14	.230
Mixed		2.38	1.73–3.27	<.001	1.69		1.27–2.27	<.001
Hispanic/Latino (reference ‐ no)	21,298							
Yes		1.90	1.52–2.37	<.001	0.93		0.75–1.16	.513
Education (reference – attended college or higher)	21,299							
High school or lower		2.68	2.28–3.15	<.001	1.83		1.57–2.13	<.001
Marital status (reference ‐ with partner[s])	21,296							
Without partner(s)		3.01	2.58–3.51	<.001	1.69		1.45–1.98	<.001
Employment status (reference – not working)	21,287			<.001				<.001
Government		0.62	0.48–0.80	<.001	0.59		0.45–0.77	<.001
Private		1.33	1.14–1.56	<.001	1.13		0.95–1.34	.153
Self‐employed		1.48	1.12–1.96	.006	1.43		1.09–1.88	.009
# of household members	20,703	1.17	1.11–1.23	<.001	1.07		1.02–1.13	.010
Public plan (reference ‐ not covered)	21,299							
Covered by public plan(s)		0.62	0.53–0.72	<.001	0.47		0.39–0.57	<.001
Private plan (reference ‐ not covered)	21,245							
Covered by private plan(s)		0.35	0.31–0.41	<.001	0.32		0.27–0.37	<.001
Hypertension (reference ‐ no)	21,299							
Yes		0.81	0.69–0.94	.007		1.19	1.01–1.40	.035
Diabetes (reference ‐ no)	21,299							
Yes		1.43	1.17–1.74	<.001		1.85	1.52–2.25	<.001
Chronic lung disease (reference ‐ no)	21,299							
Yes		2.43	1.80–3.29	<.001		2.06	1.54–2.74	<.001
Mental health conditions (reference ‐ no)	21,299							
Yes		4.60	3.91–5.40	<.001		2.74	2.35–3.19	<.001

Abbreviation: OOP:HHI, out‐of‐pocket cost burden relative to household income.

^a^ Random intercepts incorporated to account for responses from the same respondents.

^b^ Statistically significant variables in univariable analyses were included in multivariable analysis.

Additionally, female sex, lower education attainment, and unpartnered status were associated with a higher risk of cost‐related care avoidance (all *p* < .001), whereas older age, health insurance coverage, and current employment reduced the odds of cost‐related care avoidance (all *p* < .001) in the multivariable model. Having hypertension, diabetes, chronic lung disease, or mental health issues as a comorbid condition was also associated with an increased odds of cost‐related care avoidance (all *p* < .05) (Table 2).

### Classification performance of OOP:HHI alone and multivariable model

**FIGURE 4:**
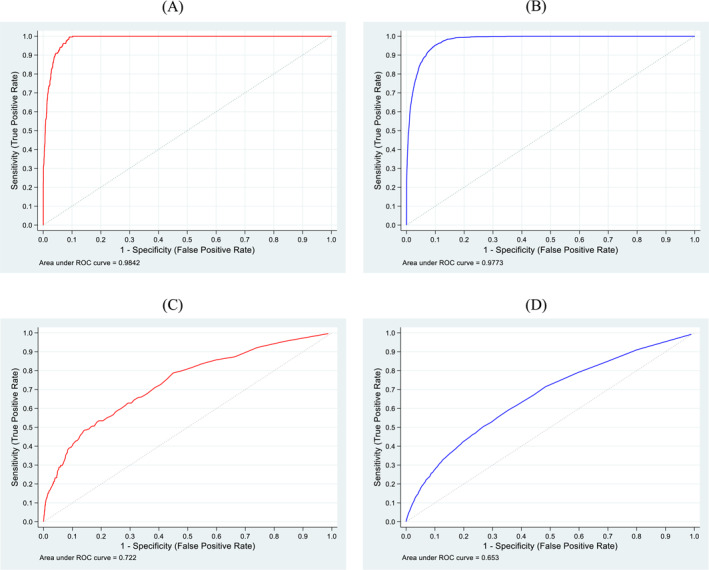
ROC curve of multivariable model in (A) cancer subgroup and (B) noncancer subgroup and OOP:HHI alone in (C) cancer subgroup and (D) noncancer subgroup. OOP:HHI (in %) is a continuous variable. OOP:HHI indicates out‐of‐pocket cost burden relative to household income; ROC, receiver operating characteristic.

The optimal threshold for OOP:HHI alone was 1.3% for the cancer subgroup and 0.8% for the noncancer subgroup, far below the conventional thresholds used to determine catastrophic healthcare expenditure with a sensitivity/specificity of 66%/61%, respectively (Supplemental Figure 1). In contrast, at the optimal threshold of predicted probabilities from the multivariable model, the sensitivity/specificity was 94%/93% for the cancer and noncancer subgroup, respectively (Supplemental Figure 2). Consistent with these findings, the AUC of the ROC for the multivariable model was 0.98 for the cancer subgroup (Figure 4A) and 0.98 for the noncancer subgroup (Figure 4B), whereas the AUC of the ROC for OOP:HHI alone was 0.72 for the cancer subgroup (Figure 4C) and 0.65 for the noncancer subgroup (Figure 4D).

## DISCUSSION

In this large‐scale observational study, we found that OOP:HHI was positively associated with the odds of cost‐related care avoidance. Although this association was attenuated among respondents with cancer history in the unadjusted analysis, the interaction effect was no longer significant after adjusting for other respondent characteristics. Importantly, we found that OOP:HHI alone is a poor predictor of cost‐related care avoidance at the individual level, although this measure is widely used for policy evaluation. Incorporating respondent characteristics substantially improved classification performance, highlighting the importance of accounting for factors beyond cost burden alone when predicting risk of cost‐related care avoidance.

Our findings build on a limited but growing body of literature examining the relationship between OOP medical costs and health‐seeking behavior among cancer survivors. A recent systematic literature review found few studies addressing this question with a wide variation in how health‐seeking behavior was measured.^12^ Among these, Guy et al. reported that U.S. cancer survivors spending >20% of annual family income on OOP health care costs were significantly more likely to report the inability to obtain medical care, dental care, or prescription medications because of cost and delayed necessary care, compared with those spending <20%.^16^ These observations align with our findings that cost‐related care avoidance among cancer patients increased sharply once OOP spending surpasses specific income‐based thresholds. Similarly, prior analyses using the National Health Interview Survey found that cancer survivors were significantly more likely than respondents without cancer history to experience financial barriers to care including forgone medical care.^17^ However, in our adjusted models, cancer history was not independently associated with cost‐related care avoidance. This likely reflects the incorporation of capacity‐to‐pay measures and comorbidities, both of which influence the risk of cost‐related care avoidance and may mediate the apparent difference between cancer and noncancer populations. The proportion of respondents exceeding conventional OOP:HHI thresholds in our study was also lower than in prior cancer‐focused studies,^18^, ^19^, ^20^, ^21^ likely reflecting differences in study populations, study periods (pre‐ vs. post‐Affordable Care Act), OOP cost definitions, and income denominators.

In our unadjusted analyses, we observed that the likelihood of cost‐related care avoidance increased more sharply with rising OOP:HHI among respondents without cancer history compared with those with cancer history. This pattern may reflect a higher degree of price inelasticity of health care demand among individuals with cancer history, who may prioritize health care spending because of their prior experience with a life‐threatening condition. Although this interaction was attenuated in adjusted models, the broader implication is that applying uniform OOP:HHI thresholds across disease areas may not fully reflect differences in health care prioritization or patient characteristics. Although this may not be a critical issue in policy evaluation, where the key aim is to evaluate the population‐level impact of interventions on the financial status of households and/or individuals, caution should be taken before using the same OOP:HHI thresholds to predict cost‐related care avoidance in different disease areas. Alternatively, as our findings have suggested, a multivariable risk‐stratification approach should be adopted to adjust for the potential influence of disease history on price elasticity or varying patient characteristics.

Conventionally, 10%, 25%, and 40% have been used as the OOP:HHI thresholds to indicate catastrophic health care expenditure for policy evaluation.^10^ However, our study indicated that most individuals, regardless of cancer history, incur much lower OOP:HHI levels and may still report cost‐related care avoidance. The optimal OOP:HHI thresholds for identifying individuals at risk of cost‐related care avoidance was well below the traditional 10% benchmark, but even at these lower thresholds, sensitivity and specificity remained modest (approximately 60%–70%) for both the cancer and noncancer subgroups. Raising the threshold increased specificity at the cost of sharply reduced sensitivity, illustrating that higher thresholds will fail to capture a substantial proportion of patients experiencing financial toxicity. In contrast, a predictive model that incorporated OOP:HHI along with sociodemographic and clinical characteristics achieved substantially higher sensitivity and specificity (94%), aligning with ROC diagnostics that favored the multivariable approach over the use of OOP:HHI alone. These findings suggest that using OOP cost‐based tools to identify patients at elevated risk of cost‐related care avoidance should incorporate other routinely collected information from electronic health records rather than relying solely on OOP medical expenditure.

OOP costs have long been recognized as important in medical decision‐making. In a survey of medical oncologists, 84% reported that OOP costs affected treatment recommendations, and 16% acknowledged omitting specific treatment options based on cost‐related concerns.^22^, ^23^ Despite this strong awareness, many oncologists remain uncertain how the cost of care should influence their treatment recommendations and feel uncomfortable discussing cost issues with patients.^24^, ^25^, ^26^ Our findings reinforce the importance of routinely discussing cost‐related issues when identifying treatment options to ensure adequate adherence to care and integrating routine financial toxicity screening and electronic health record–embedded risk‐stratification algorithms into clinical workflows to proactively identify at‐risk patients. By empirically mapping the relationship between OOP burden and cost‐related care avoidance or other adverse health‐seeking behavior such as nonadherence to treatment, system‐level tools leveraging OOP medical expenditure data can be developed to identify patients who may benefit from extensive discussion and targeted interventions. Health systems could leverage risk‐stratification tools to target outreach and benefit redesign toward enrollees most likely to forgo needed care.

## LIMITATIONS

Our study has several limitations. First, the retrospective observational nature of the study limits our ability to draw causal conclusions. Reverse causation cannot be entirely ruled out because of the cross‐sectional study design. The intent of this analysis, however, is to characterize associations and describe OOP cost burden and cost‐related care avoidance within this population. Cross‐sectional estimates provide essential context to inform the design of future longitudinal studies. Second, the use of self‐reported measures to indicate cost‐related care avoidance introduces the potential for recall or social desirability bias. However, these measures are widely used in national surveys and have demonstrated strong validity, particularly for coping behaviors such as delaying or skipping needed care. There is also no gold‐standard objective measure. In addition, the survey did not capture clinical information, such as cancer staging, tumor site, and treatment history, which may influence OOP cost burden and health‐seeking behavior, and other dimensions of care avoidance, including delaying treatment or nonadherence. Future studies incorporating richer clinical detail and measures of other coping strategies may further clarify the pathways linking OOP burden to health care–seeking behavior.

## CONCLUSIONS

Our study demonstrated that higher OOP:HHI was associated with greater odds of cost‐related care avoidance among individuals with and without cancer history, with similar trends of association in both subgroups. OOP:HHI alone showed limited ability to identify individuals at risk of cost‐related care avoidance and the optimal threshold was well below conventional catastrophic expenditure cutoffs and were only modestly accurate. In contrast, models incorporating other patient characteristics demonstrated substantially higher classification performance, indicating that cost burden alone is insufficient for risk stratification. Together, these findings support developing system‐level risk‐stratification tools that integrate OOP:HHI with routinely collected patient characteristics to proactively identify individuals at risk of financial toxicity and guide targeted interventions.

## AUTHOR CONTRIBUTIONS

**Xi Liang:** Conceptualization; data curation; formal analysis; investigation; methodology; visualization; writing—original draft preparation; and writing—review & editing. **Djin Tay:** Conceptualization; investigation and writing—review & editing. **Yu Ke:** Conceptualization; investigation; and writing—review & editing. **Ding Quan Ng:** Investigation and writing—review & editing. **Chanthawat Patikorn:** Investigation and writing—review & editing. **Carl Asche:** Investigation and writing—review & editing. **Nathorn Chaiyakunapruk:** Conceptualization; investigation; and writing—review & editing. **Anne C. Kirchhoff:** Conceptualization; investigation; and writing—review & editing. **Raymond Javan Chan:** Conceptualization; investigation; and writing—review & editing. **Alexandre Chan:** Conceptualization; investigation; and writing—review & editing. **Chia Jie Tan:** Conceptualization; data curation; formal analysis; investigation; methodology; project administration; resources; supervision; writing—original draft preparation; and writing—review & editing.

## CONFLICT OF INTEREST STATEMENT

The authors declare no conflict of interest.

## FUNDING

None.

## IRB STATEMENT

This study was exempted from ethical review by the University of Utah Institutional Review Board on August 1, 2024 (IRB_00180273). Informed consent was waived due to the retrospective design of the study and deidentified data for analysis.

## Supporting information

Supplementary Material

## Data Availability

The data that support the findings of this study are available from Center for Economic and Social Research, USC. Restrictions apply to the availability of these data, which were used under license for this study. Data are available from https://uasdata.usc.edu/page/Data+Products with the permission of Center for Economic and Social Research, USC.
