# Supplementary material for: Comparing the association between out‐of‐pocket cost burden and cost‐related care avoidance among individuals with and without a history of cancer
Source: Cancer. 2026 Jun 15;132(12):e70495. doi: 10.1002/cncr.70495 (PMC13266579; doi:10.1002/cncr.70495)
Supplement: Supplementary file 1 — Supplementary Material [file CNCR-132-e70495-s001.docx]

**Supplementary Materials**

**Supplemental Table 1. Distribution of OOP:HHI stratified by cancer history and cost-related care avoidance**

| **Category** | **All responses**  **(N = 21299)** | **Cancer history**  **(N = 2180)** | **No cancer history**  **(N = 19119)** | **No care-related care avoidance**  **(N = 18015)** | **Indicated care-related care avoidance**  **(N = 3284)** |
| --- | --- | --- | --- | --- | --- |
| 0% | 223 (1.1%) | 25 (1.2%) | 198 (1.0%) | 198 (1.1%) | 25 (0.8%) |
| >0% to <0.1% | 3572 (16.8%) | 302 (13.9%) | 3270 (17.1%) | 3314 (18.4%) | 258 (7.9%) |
| >0.1% to <0.5% | 6198 (29.1%) | 573 (26.3%) | 5625 (29.4%) | 5582 (31.0%) | 616 (18.8%) |
| >0.5% to <1% | 3119 (14.6%) | 309 (14.2%) | 2810 (14.7%) | 2661 (14.8%) | 458 (14.0%) |
| >1% to <5% | 6117 (28.7%) | 703 (32.3%) | 5414 (28.3%) | 4964 (27.6%) | 1153 (35.1%) |
| >5% to <10% | 1143 (5.4%) | 157 (7.2%) | 986 (5.2%) | 769 (4.3%) | 374 (11.4%) |
| >10% to <25% | 637 (3.0%) | 67 (3.1%) | 570 (3.0%) | 390 (2.2%) | 247 (7.5%) |
| >25% to <40% | 112 (0.5%) | 13 (0.6%) | 99 (0.5%) | 59 (0.3%) | 53 (1.6%) |
| >40 | 178 (0.8%) | 31 (1.4%) | 147 (0.8%) | 78 (0.4%) | 100 (3.1%) |

**Supplemental Table 2. Association between OOP:HHI (as a categorical variable with 10% as threshold) and cost-related care avoidance (N = 10,811)**

| **Variables** | **Adjusted (multivariable) analysis^*,∆^** | | |  |
| --- | --- | --- | --- | --- |
|  | **Responses = 20,533** | | |  |
|  | **Odds ratio** | **95% CI** | **p-value** |  |
| OOP:HHI (in %) > 10%^#^ (reference - no) |  |  |  |  |
| Yes | 4.36 | 3.36, 5.68 | < 0.001 |  |
| Cancer (reference – without cancer history) |  |  |  |  |
| With cancer history | 0.78 | 0.58, 1.03 | 0.081 |  |
| Cancer interaction (reference - without cancer history) |  |  |  |  |
| With cancer history | 1.41 | 0.66, 3.02 | 0.381 |  |
| Age at survey | 0.96 | 0.95, 0.96 | < 0.001 |  |
| Gender (reference - female) |  |  |  |  |
| Male | 0.55 | 0.47, 0.64 | < 0.001 |  |
| Race (reference - White) |  |  | 0.004 |  |
| Black only | 1.14 | 0.88, 1.48 | 0.332 |  |
| American Indian or Alaska Native only | 1.23 | 0.72, 2.12 | 0.445 |  |
| Asian and Pacific Islander only | 0.82 | 0.59, 1.14 | 0.239 |  |
| Mixed | 1.68 | 1.25, 2.25 | < 0.001 |  |
| Hispanic/Latino (reference - no) |  |  |  |  |
| Yes | 0.93 | 0.75, 1.16 | 0.536 |  |
| Education (reference – attended college or higher) |  |  |  |  |
| High school or lower | 1.81 | 1.56, 2.11 | < 0.001 |  |
| Marital status (reference - with partner[s]) |  |  |  |  |
| Without partner(s) | 1.69 | 1.45, 1.98 | < 0.001 |  |
| Employment status (reference – not working) |  |  | < 0.001 |  |
| Government | 0.59 | 0.46, 0.77 | < 0.001 |  |
| Private | | 1.14 | 0.96, 1.36 | 0.122 |
| Self-employed | 1.44 | 1.10, 1.89 | 0.008 |  |
| # of household members | 1.08 | 1.02, 1.14 | 0.006 |  |
| Public plan (reference - not covered) |  |  |  |  |
| Covered by public plan(s) | 0.47 | 0.39, 0.56 | < 0.001 |  |
| Private plan (reference - not covered) |  |  |  |  |
| Covered by private plan(s) | 0.32 | 0.27, 0.37 | < 0.001 |  |
| Hypertension (reference - no) | |  |  |  |
| Yes | | 1.18 | 1.01, 1.39 | 0.042 |
| Diabetes (reference - no) | |  |  |  |
| Yes | | 1.85 | 1.52, 2.26 | < 0.001 |
| Chronic lung disease (reference - no) | |  |  |  |
| Yes | | 2.04 | 1.53, 2.73 | < 0.001 |
| Mental health conditions (reference - no) | |  |  |  |
| Yes | | 2.74 | 2.35, 3.20 | < 0.001 |

*# OOP:HHI is a binary variable with 10% as a cut-off*

** Random intercepts incorporated to account for responses from the same respondents*

*∆ Statistically significant variables in univariable analyses were included in multivariable analysis*

**Supplemental Table 3. Association between OOP:HHI (as a categorical variable with 25% as threshold) and cost-related care avoidance (N = 10,811)**

| **Variables** | **Adjusted (multivariable) analysis^*,∆^** | | |  |
| --- | --- | --- | --- | --- |
|  | **Responses = 20,533** | | |  |
|  | **Odds ratio** | **95% CI** | **p-value** |  |
| OOP:HHI (in %) > 25%^#^ (reference - no) |  |  |  |  |
| Yes | 5.23 | 3.30, 8.30 | < 0.001 |  |
| Cancer (reference – without cancer history) |  |  |  |  |
| With cancer history | 0.77 | 0.58, 1.01 | 0.063 |  |
| Cancer interaction (reference - without cancer history) |  |  |  |  |
| With cancer history | 2.37 | 0.74, 7.61 | 0.149 |  |
| Age at survey | 0.96 | 0.95, 0.96 | < 0.001 |  |
| Gender (reference - female) |  |  |  |  |
| Male | 0.54 | 0.46, 0.63 | < 0.001 |  |
| Race (reference - White) |  |  | 0.004 |  |
| Black only | 1.12 | 0.86, 1.46 | 0.420 |  |
| American Indian or Alaska Native only | 1.25 | 0.73, 2.15 | 0.418 |  |
| Asian and Pacific Islander only | 0.82 | 0.59, 1.14 | 0.243 |  |
| Mixed | 1.71 | 1.28, 2.29 | < 0.001 |  |
| Hispanic/Latino (reference - no) |  |  |  |  |
| Yes | 0.93 | 0.75, 1.16 | 0.523 |  |
| Education (reference – attended college or higher) |  |  |  |  |
| High school or lower | 1.84 | 1.58, 2.15 | < 0.001 |  |
| Marital status (reference - with partner[s]) |  |  |  |  |
| Without partner(s) | 1.71 | 1.46, 2.00 | < 0.001 |  |
| Employment status (reference – not working) |  |  | < 0.001 |  |
| Government | 0.58 | 0.44, 0.75 | < 0.001 |  |
| Private | | 1.12 | 0.94, 1.33 | 0.192 |
| Self-employed | 1.42 | 1.08, 1.86 | 0.012 |  |
| # of household members | 1.07 | 1.02, 1.13 | 0.011 |  |
| Public plan (reference - not covered) |  |  |  |  |
| Covered by public plan(s) | 0.46 | 0.39, 0.56 | < 0.001 |  |
| Private plan (reference - not covered) |  |  |  |  |
| Covered by private plan(s) | 0.31 | 0.27, 0.36 | < 0.001 |  |
| Hypertension (reference - no) | |  |  |  |
| Yes | | 1.20 | 1.02, 1.42 | 0.025 |
| Diabetes (reference - no) | |  |  |  |
| Yes | | 1.87 | 1.53, 2.28 | < 0.001 |
| Chronic lung disease (reference - no) | |  |  |  |
| Yes | | 2.05 | 1.54, 2.75 | < 0.001 |
| Mental health conditions (reference - no) | |  |  |  |
| Yes | | 2.79 | 2.40, 3.26 | < 0.001 |

*# OOP:HHI is a binary variable with 25% as a cut-off*

** Random intercepts incorporated to account for responses from the same respondents*

*∆ Statistically significant variables in univariable analyses were included in multivariable analysis*

**Supplemental Table 4. Association between OOP:HHI (as a categorical variable with 40% as threshold) and cost-related care avoidance (N = 10,811)**

| **Variables** | **Adjusted (multivariable) analysis^*,∆^** | | |  |
| --- | --- | --- | --- | --- |
|  | **Responses = 20,533** | | |  |
|  | **Odds ratio** | **95% CI** | **p-value** |  |
| OOP:HHI (in %) > 40%^#^ (reference - no) |  |  |  |  |
| Yes | 5.10 | 2.77, 9.39 | < 0.001 |  |
| Cancer (reference – without cancer history) |  |  |  |  |
| With cancer history | 0.77 | 0.58, 1.02 | 0.065 |  |
| Cancer interaction (reference - without cancer history) |  |  |  |  |
| With cancer history | 3.51 | 0.80, 15.36 | 0.096 |  |
| Age at survey | 0.95 | 0.95, 0.96 | < 0.001 |  |
| Gender (reference - female) |  |  |  |  |
| Male | 0.53 | 0.46, 0.63 | < 0.001 |  |
| Race (reference - White) |  |  | 0.003 |  |
| Black only | 1.11 | 0.85, 1.45 | 0.425 |  |
| American Indian or Alaska Native only | 1.25 | 0.73, 2.15 | 0.421 |  |
| Asian and Pacific Islander only | 0.83 | 0.59, 1.15 | 0.259 |  |
| Mixed | 1.72 | 1.28, 2.30 | < 0.001 |  |
| Hispanic/Latino (reference - no) |  |  |  |  |
| Yes | 0.94 | 0.75, 1.16 | 0.555 |  |
| Education (reference – attended college or higher) |  |  |  |  |
| High school or lower | 1.85 | 1.59, 2.15 | < 0.001 |  |
| Marital status (reference - with partner[s]) |  |  |  |  |
| Without partner(s) | 1.71 | 1.46, 2.00 | < 0.001 |  |
| Employment status (reference – not working) |  |  | < 0.001 |  |
| Government | 0.57 | 0.44, 0.74 | < 0.001 |  |
| Private | | 1.10 | 0.93, 1.31 | 0.262 |
| Self-employed | 1.41 | 1.08, 1.86 | 0.013 |  |
| # of household members | 1.07 | 1.01, 1.13 | 0.013 |  |
| Public plan (reference - not covered) |  |  |  |  |
| Covered by public plan(s) | 0.47 | 0.39, 0.56 | < 0.001 |  |
| Private plan (reference - not covered) |  |  |  |  |
| Covered by private plan(s) | 0.31 | 0.26, 0.36 | < 0.001 |  |
| Hypertension (reference - no) | |  |  |  |
| Yes | | 1.21 | 1.03, 1.42 | 0.024 |
| Diabetes (reference - no) | |  |  |  |
| Yes | | 1.86 | 1.53, 2.27 | < 0.001 |
| Chronic lung disease (reference - no) | |  |  |  |
| Yes | | 2.08 | 1.56, 2.78 | < 0.001 |
| Mental health conditions (reference - no) | |  |  |  |
| Yes | | 2.80 | 2.40, 3.26 | < 0.001 |

*# OOP:HHI is a binary variable with 40% as a cut-off*

** Random intercepts incorporated to account for responses from the same respondents*

*∆ Statistically significant variables in univariable analyses were included in multivariable analysis*

**Supplemental Table 5. Association between OOP:HHI (in % as a continuous variable) and cost-related care avoidance in respondents aged < 65 years old (N = 8,787)**

| **Variables** | **Adjusted (multivariable) analysis^*,∆^** | | |  |
| --- | --- | --- | --- | --- |
|  | **Responses = 15,524** | | |  |
|  | **Odds ratio** | **95% CI** | **p-value** |  |
| OOP:HHI (in %) | 1.03 | 1.02, 1.04 | < 0.001 |  |
| Cancer (reference – without cancer history) |  |  |  |  |
| With cancer history | 0.98 | 0.71, 1.36 | 0.901 |  |
| Cancer interaction (reference - without cancer history) |  |  |  |  |
| With cancer history | 0.99 | 0.97, 1.00 | 0.072 |  |
| Age at survey | 0.97 | 0.97, 0.98 | < 0.001 |  |
| Gender (reference - female) |  |  |  |  |
| Male | 0.55 | 0.47, 0.65 | < 0.001 |  |
| Race (reference - White) |  |  | 0.015 |  |
| Black only | 1.00 | 0.76, 1.32 | 0.983 |  |
| American Indian or Alaska Native only | 0.91 | 0.52, 1.58 | 0.729 |  |
| Asian and Pacific Islander only | 0.81 | 0.58, 1.13 | 0.216 |  |
| Mixed | 1.62 | 1.20, 2.18 | 0.002 |  |
| Hispanic/Latino (reference - no) |  |  |  |  |
| Yes | 0.96 | 0.77, 1.19 | 0.720 |  |
| Education (reference – attended college or higher) |  |  |  |  |
| High school or lower | 1.74 | 1.48, 2.04 | < 0.001 |  |
| Marital status (reference - with partner[s]) |  |  |  |  |
| Without partner(s) | 1.73 | 1.47, 2.04 | < 0.001 |  |
| Employment status (reference – not working) |  |  | < 0.001 |  |
| Government | 0.52 | 0.40, 0.67 | < 0.001 |  |
| Private | | 1.02 | 0.86, 1.22 | 0.789 |
| Self-employed | 1.25 | 0.95, 1.66 | 0.114 |  |
| # of household members | 1.06 | 1.00, 1.12 | 0.034 |  |
| Public plan (reference - not covered) |  |  |  |  |
| Covered by public plan(s) | 0.64 | 0.53, 0.79 | < 0.001 |  |
| Private plan (reference - not covered) |  |  |  |  |
| Covered by private plan(s) | 0.30 | 0.25, 0.36 | < 0.001 |  |
| Hypertension (reference - no) | |  |  |  |
| Yes | | 1.22 | 1.03, 1.44 | 0.022 |
| Diabetes (reference - no) | |  |  |  |
| Yes | | 1.58 | 1.27, 1.96 | < 0.001 |
| Chronic lung disease (reference - no) | |  |  |  |
| Yes | | 2.06 | 1.49, 2.85 | < 0.001 |
| Mental health conditions (reference - no) | |  |  |  |
| Yes | | 2.55 | 2.18, 2.99 | < 0.001 |

** Random intercepts incorporated to account for responses from the same respondents*

*∆ Statistically significant variables in univariable analyses were included in multivariable analysis*

**Supplemental Table 6. Association between OOP:HHI (in % as a continuous variable) and cost-related care avoidance by adjusting for survey start year (as a continuous variable) (N = 10,811)**

| **Variables** | **Adjusted (multivariable) analysis^*,∆^** | | |  |
| --- | --- | --- | --- | --- |
|  | **Responses = 20,533** | | |  |
|  | **Odds ratio** | **95% CI** | **p-value** |  |
| OOP:HHI (in %) | 1.03 | 1.02, 1.03 | < 0.001 |  |
| Cancer (reference – without cancer history) |  |  |  |  |
| With cancer history | 0.82 | 0.62, 1.09 | 0.175 |  |
| Cancer interaction (reference - without cancer history) |  |  |  |  |
| With cancer history | 0.99 | 0.98, 1.01 | 0.403 |  |
| Age at survey | 0.96 | 0.95, 0.96 | < 0.001 |  |
| Gender (reference - female) |  |  |  |  |
| Male | 0.54 | 0.46, 0.63 | < 0.001 |  |
| Race (reference - White) |  |  | 0.005 |  |
| Black only | 1.15 | 0.88, 1.50 | 0.320 |  |
| American Indian or Alaska Native only | 1.25 | 0.72, 2.16 | 0.422 |  |
| Asian and Pacific Islander only | 0.86 | 0.62, 1.20 | 0.371 |  |
| Mixed | 1.71 | 1.27, 2.29 | < 0.001 |  |
| Hispanic/Latino (reference - no) |  |  |  |  |
| Yes | 0.96 | 0.77, 1.20 | 0.720 |  |
| Education (reference – attended college or higher) |  |  |  |  |
| High school or lower | 1.79 | 1.53, 2.08 | < 0.001 |  |
| Marital status (reference - with partner[s]) |  |  |  |  |
| Without partner(s) | 1.72 | 1.47, 2.01 | < 0.001 |  |
| Employment status (reference – not working) |  |  | < 0.001 |  |
| Government | 0.60 | 0.46, 0.77 | < 0.001 |  |
| Private | | 1.15 | 0.97, 1.37 | 0.112 |
| Self-employed | 1.46 | 1.11, 1.91 | 0.007 |  |
| # of household members | 1.07 | 1.02, 1.13 | 0.009 |  |
| Public plan (reference - not covered) |  |  |  |  |
| Covered by public plan(s) | 0.47 | 0.39, 0.57 | < 0.001 |  |
| Private plan (reference - not covered) |  |  |  |  |
| Covered by private plan(s) | 0.31 | 0.26, 0.36 | < 0.001 |  |
| Hypertension (reference - no) | |  |  |  |
| Yes | | 1.20 | 1.02, 1.41 | 0.029 |
| Diabetes (reference - no) | |  |  |  |
| Yes | | 1.89 | 1.55, 2.31 | < 0.001 |
| Chronic lung disease (reference - no) | |  |  |  |
| Yes | | 2.04 | 1.52, 2.73 | < 0.001 |
| Mental health conditions (reference - no) | |  |  |  |
| Yes | | 2.87 | 2.45, 3.35 | < 0.001 |
| Survey year | | 0.95 | 0.92, 0.97 | 0.026 |

** Random intercepts incorporated to account for responses from the same respondents*

*∆ Statistically significant variables in univariable analyses were included in multivariable analysis*

**Supplemental Figure 1. Sensitivity & specificity of OOP:HHI alone in (A) cancer subgroup and (B) non-cancer subgroup**

(A)

(B)

** OOP:HHI (in %) is a continuous variable*

**Supplemental Figure 2. Sensitivity and specificity of multivariable model in (A) cancer subgroup and (B) non-cancer subgroup**

(A)

(B)

** OOP:HHI (in %) is a continuous variable*
